# Endothelial Dysfunction in Patients with Severe Mitral Regurgitation

**DOI:** 10.3390/jcm8060835

**Published:** 2019-06-12

**Authors:** Benedetta Porro, Paola Songia, Veronika A. Myasoedova, Vincenza Valerio, Donato Moschetta, Paola Gripari, Laura Fusini, Laura Cavallotti, Paola Canzano, Linda Turnu, Francesco Alamanni, Marina Camera, Viviana Cavalca, Paolo Poggio

**Affiliations:** 1 Centro Cardiologico Monzino, I.R.C.C.S., 20138 Milan, Italy; benedetta.porro@ccfm.it (B.P.); paola.songia@ccfm.it (P.S.); veronika.myasoedova@ccfm.it (V.A.M.); vincenza.valerio@ccfm.it (V.V.); donato.moschetta@ccfm.it (D.M.); paola.gripari@ccfm.it (P.G.); laura.fusini@ccfm.it (L.F.); laura.cavallotti@ccfm.it (L.C.); paola.canzano@ccfm.it (P.C.); linda.1987@libero.it (L.T.); francesco.alamanni@ccfm.it (F.A.); marina.camera@ccfm.it (M.C.); viviana.cavalca@ccfm.it (V.C.); 2 Dipartimento di Medicina Clinica e Chirurgia, Università degli Studi di Napoli Federico II, 80138 Naples, Italy; 3 Department of Pharmacological and Biomolecular Sciences, Università degli Studi di Milano, 20133 Milan, Italy

**Keywords:** glutathione, osteoprotegerin, oxidative stress, endothelial microparticles, mitral valve prolapse

## Abstract

Mitral valve prolapse (MVP) is the most common cause of severe mitral regurgitation. It has been reported that MVP patients—candidates for mitral valve repair (MVRep)—showed an alteration in the antioxidant defense systems as well as in the L-arginine metabolic pathway. In this study, we investigate if oxidative stress and endothelial dysfunction are an MVP consequence or driving factors. Forty-five patients undergoing MVRep were evaluated before and 6 months post surgery and compared to 29 controls. Oxidized (GSSG) and reduced (GSH) forms of glutathione, and L-arginine metabolic pathway were analyzed using liquid chromatography-tandem mass spectrometry methods while osteoprotegerin (OPG) through the ELISA kit and circulating endothelial microparticles (EMP) by flow cytometry. Six-month post surgery, in MVP patients, the GSSG/GSH ratio decreased while symmetric and asymmetric dimethylarginines levels remained comparable to the baseline. Conversely, OPG levels significantly increased when compared to their baseline. Finally, pre-MVRep EMP levels were significantly higher in patients than in controls and did not change post surgery. Overall, these results highlight that MVRep completely restores the increased oxidative stress levels, as evidenced in MVP patients. Conversely, no amelioration of endothelial dysfunction was evidenced after surgery. Thus, therapies aimed to restore a proper endothelial function before and after surgical repair could benefit MVP patients.

## 1. Introduction

Mitral valve prolapse (MVP) is a debilitating disease afflicting 2% to 3% of people [1,2]. Even though the pathology was first described in the late 1800s [1], no major risk factor has been identified yet [3]. In addition, despite the fact that several studies have evaluated the effectiveness of angiotensin-converting enzyme inhibitors [4,5] and beta-blockers [6,7], no recommended pharmacological therapies for severe mitral regurgitation (MR) with associated MVP have been found. To date, the diagnostic gold standard is two dimensional (2D) echocardiography [8,9,10], whereas surgical intervention is the therapeutic choice when the prolapse causes symptomatic severe regurgitation [11].

The mitral valve has a complex architectural structure, and its maintenance is regulated by valve interstitial cells, directly producing, secreting, and degrading the extracellular matrix [1]. In addition, the monolayer of valve endothelial cells, covering the entire surface of the leaflets, plays an important role in the homeostasis of the interstitial cells [12]. Thus, when endothelial dysfunction occurs, this tight regulation is disrupted, leading to MVP [5,6].

In this context, we previously reported that MVP patients with severe regurgitation showed an impairment in the L-arginine (Arg)/nitric oxide (NO) biosynthetic pathway when compared to healthy subjects [13]. Furthermore, it has also been demonstrated that endothelial microparticles (EMP) increase in mitral valve disease and impair mitral valve endothelial function [14], supporting the role of these subcellular fragments as surrogate markers of endothelial dysfunction [15].

MVP is primarily caused by myxomatous mitral valve degeneration, a process characterized by an increment of the thickness of valve leaflets and the loss of their well-organized structure. This process is directly caused by proteoglycans accumulation and structural alteration of collagen [16]. These alterations could be caused by transforming growth factor-b superfamily proteins and by a weakening of the antioxidant defense systems [17,18,19].

Previously, we have shown altered systemic oxidative stress homeostasis as well as increased osteoprotegerin (OPG) plasma levels in MVP patients when compared to controls [20]. In particular, circulating OPG and oxidative stress status were positively associated with severe mitral regurgitation due to MVP. Based on these premises, we developed a multivariable regression model, combining OPG with oxidative stress markers, which were able to discriminate MVP patients from healthy subjects with high accuracy and precision [20]. These results are in accordance with the pivotal role played by OPG during the endothelial-to-mesenchymal transition (EndMT) [21], a process known to be linked to MVP [22,23]. In particular, endothelial cells, during EndMT, increased production and secretion of OPG that in turn contributed to the extracellular matrix alterations involved in MVP progression. These data underlined OPG contribution in disease progression by interfering with the correct valve endothelial function [21].

Indeed, it has been hypothesized that both alterations in oxidative stress status and in endothelial function were associated with MR; however, up to now no data regarding these processes in MVP patients after surgical restoration of normal valve function is available.

Given this background, the aim of the present study is to investigate if surgical intervention is able to restore, to some extent, the systemic alterations evidenced in MVP patients before surgery, elucidating if oxidative stress and endothelial dysfunction are an MVP consequence or the driving factors.

## 2. Experimental Section

### 2.1. Patient Demographics

This prospective observational study was approved by the IRCCS Centro Cardiologico Monzino (CCM) Institutional Review Board and by the IRCCS Istituto Europeo di Oncologia and CCM Ethics Committee. The investigation conformed to the principles outlined in the Declaration of Helsinki (1964) and informed consent was obtained from all the participants. Over one year period (June 2016–June 2017), 45 patients undergoing mitral valve repair (MVRep), due to primary posterior MVP with severe MR, were enrolled. The preoperative inclusion criteria were the need for elective, isolated surgical procedure, over 18 year of age, ejection fraction >30%, normal sinus rhythm, and no history of atrial fibrillation. Patients with premature menopause and/or osteoporosis, secondary MVP, previous aortic or mitral valve surgery, rheumatic heart disease, endocarditis, active malignancy, chronic liver failure, calcium regulation disorders (hyperparathyroidism, hyperthyroidism, and hypothyroidism), and chronic or acute inflammatory states (corticosteroid intake, sepsis, autoimmune disease, inflammatory bowel disease, and c-reactive protein >5.0 mg/L, respectively) were excluded. To make a comparison, during the same time-period we also enrolled 29 controls with cardiovascular risk factors similar to MVP patients, selected from those attending the clinic for global control of cardiovascular risk at CCM. For controls’ selection, we took into account the following cardiovascular risk factors: age, sex, diabetes, hypertension, hypercholesterolemia, and smoking habits. All subjects were assessed with detailed medical history, physical examination, and echocardiography. In all patients, blood collection was performed before coronary angiography with the exception of the controls that underwent sample collection at a scheduled visit.

### 2.2. Echocardiography Evaluation

Two-dimensional (2D) echocardiography represents the gold standard for diagnosis and assessment of MVP patients [9,24]. The combination of 2D and 3D echocardiographic imaging provides detailed morphological and functional assessment of mitral valve itself [8,25]. Experienced cardiologists of CCM performed the echocardiography scans, according to the current guideline recommendations [9,24]. Echocardiographically, MVP is defined as a single or bileaflet prolapse, at least 2 mm beyond the long-axis annular plane, while the assessment of the severity of MR relies on a multiparametric evaluation [10,26]. The included parameters are left ventricular ejection fraction (LVEF), left ventricular diastolic and systolic volumes (mL), left atrial area (cm^2^), pulmonary artery systolic pressure (PAP), and effective regurgitant orifice area (EROA).

### 2.3. Blood Sampling

Whole blood: 6 mL of peripheral blood sample was drawn from patients and controls while fasting, into tubes containing EDTA (9.3 mM; Vacutainer Systems, Becton Dickinson, Franklin Lakes, NJ, USA) kept on ice. Two hundred fifty µL of whole blood was immediately precipitated with 250 µL of 10% trichloroacetic acid (Sigma-Aldrich, St Louis, MO, USA) plus 1 mM EDTA solution. Samples were stored at −80 °C until analysis.

Plasma EDTA: anti-coagulated blood was centrifuged at 1700× *g* for 10 min at 4 °C within 30 min after being drawn. Plasma was separated, and aliquots were stored at −80 °C until analysis.

Plasma citrate: sodium citrate anti-coagulated blood was centrifuged at 1700× *g* for 10 min at 4 °C within 30 min after being drawn to obtain platelet-poor plasma (PPP). PPP was collected, and aliquots were stored at −80 °C until analysis.

The analysis of whole blood and plasma EDTA, from MVP patients and controls, was performed in a 3 months window, and all subsequent analyses were performed in duplicates in order to avoid measurement biases. A flow cytometry expert blinded from subject category (control, pre-operative MVP, or post-operative MVP) performed EMPs analysis on plasma citrate.

### 2.4. Oxidative Stress Measurement

For oxidative stress evaluation, we measured the levels of the oxidized (GSSG) and the reduced (GSH) forms of glutathione, whose ratio is a well recognized oxidative stress index (GSSG/GSH) [27]. Whole blood concentrations of GSSG and GSH were quantified using a previously developed and validated liquid chromatography-tandem mass spectrometry (LC-MS/MS) method [28]. Briefly, chromatographic separation was conducted on a Luna PFP analytical column (100 × 2.0 mm, 3 µm, Phenomenex, Torrance, CA, USA), eluted at 35°C under isocratic conditions at 200 µL/min by 1% methanol in ammonium formate 0.75 mM adjusted to pH 3.5 with formic acid. Analysis was performed by Accela chromatographic system coupled with a triple quadrupole mass spectrometer TSQ Quantum Access (Thermo Fisher Scientific, San Jose, CA, USA) using electrospray ionization source in positive ion mode. The transitions used in the multiple reaction monitoring were m/z 308.1→m/z 76.2 + 84.2 + 161.9 for GSH and m/z 613.2→m/z 230.5 + 234.6 + 354.8 for GSSG. Data were obtained after comparison with calibration curves using GSH and GSSG standard solutions (Sigma-Aldrich). The intra- and inter-day CVs % obtained with standard samples were <5% for the both the analytes considered. The limits of detection (LOD) were 0.031 µmol/L and 0.008 µmol/L for GSH and GSSG, respectively. Levels of GSH and GSSG were corrected for hemoglobin (Hb) and expressed as µmol/g Hb.

### 2.5. Antioxidants

To evaluate the antioxidant defense system, plasma vitamin E (α-tocopherol (αT) and γ-tocopherol (γT)) was measured by high-performance liquid chromatography equipped with fluorimetric detector FP-1520, after organic extraction, as previously described by Werba et al. [29]. Briefly, 100 µL of plasma sample was precipitated with ethanol 50%, and α- and γT were extracted with 1 mL of n-hexane. After evaporation to dryness under nitrogen stream of 600 µL of organic extract, the residue was dissolved in ethanol (200 µL). An aliquot (25 µL) was separated using a Discovery C18, 3.5 µm RP column (4.6 mm × 250 mm) (Supelco, College Park, GA, USA) eluted with methanol (100%) as mobile phase at flow rate of 1 mL/min. Analysis was carried out by Jasco (Tokyo, Japan) FP15-20 fluorescent detector (λecc 292 nm, λem 335 nm). ESA commercial software was used for chromatograms integration. Data were obtained after comparison with calibration curves using α- and γT pure standard solutions (Sigma-Aldrich). The intra- and the inter-day CVs %s for plasma αT were 3.3% and 4.0%, respectively, with a limit of quantification (LOQ) of 0.38 µmol/L. The corresponding values for γT were 3.3% and 4.7%, respectively, with a LOQ of 0.014 µmol/L.

### 2.6. Osteoprotegerin Evaluation

Plasma EDTA was used to measure levels of soluble OPG with an enzyme-linked immunosorbent assay (ELISA) kit (DuoSet–R&D, Minneapolis, MN, USA) following manufacturer instructions and previously validation [20]. The standard of this kit is similar to full-length OPG, making this ELISA kit more representative of circulating OPG molecule [30]. The intra- and the inter-day CVs %s for plasma OPG were 4.2% and 14.2%, respectively.

### 2.7. L-Arginine Metabolic Pathway Evaluation

For the assessment of L-arginine metabolic pathway, we simultaneously measured plasma levels of Arg, L-citrulline (Cit), L-ornithine (Orn), asymmetric (ADMA), and symmetric (SDMA) dimethylarginines by LC-MS/MS using a target metabolomic approach [31]. The ratio (Arg/ (Orn+Cit)) was calculated and used as an index of global arginine bioavailability (GABR) [32,33]. Briefly, chromatographic analysis was conducted on a Luna HILIC (hydrophilic interaction liquid chromatography) analytical column (50 × 2.0 mm, 3 µm, Phenomenex, Torrance, CA, USA). The mobile phases consisted of aqueous 1.5 mM ammonium formate (pH 3.2) (A) and 1.5 mM ammonium formate in acetonitrile/methanol (95.5:0.5, *v*/*v*) (pH 3.2) (B) at flow rate of 250 µL/min. The mobile phase gradient ran from 10% A to 70% A over 7 min, ran from 70% A to 94.5% A over 2 min, and was held at 94.5% A for 5 min, returning to 10% A over 2 min and held at 10% A for re-equilibration. The sample injection volume was 10 µL, the column temperature was set at 30 °C, and the sample injector was maintained at 10°C. Total run time per sample, including column cleaning and re-equilibration, was 25 min. Mass spectrometric analysis was performed using a TSQ Quantum Access (Thermo Fisher Scientific) triple quadrupole mass spectrometer equipped with an electrospray ionization (ESI) interface operated in positive mode. The analytes were detected by tandem mass spectrometry (MS/MS) using multiple reaction monitoring (MRM). The LOQ value was ≤0.25 µM for all compounds, making this method suitable for the analysis of samples containing relatively low concentrations of the analytes, with a satisfactory precision as documented by the intra- and inter-day CVs of less than 10%. The method was linear in a wide range of concentrations (between 0 and 20 µM), with correlation coefficients greater than 0.99 and LOD around 3–10 nM for all compounds.

### 2.8. Endothelial Microparticle (EMP) Identification

The frozen PPP was thawed at 37 °C for 3 min and then centrifuged at 13,000 g for 5 min to obtain platelet-free plasma (PFP). Sixty µL of PFP was diluted with 118 µL of 0.22 µm-pore-size-membrane-filtered PBS 1X (Gibco, Thermo Fisher Scientific) and then incubated with 2 µL of calcein AM 10 µM (Thermo Fisher Scientific) for 25 min at 37 °C in the dark in order to stain intact MPs. Afterwards, saturating concentration of an antibody against CD146, an endothelial-derived microparticles marker, conjugated with phycoerythrin (Becton Dickinson Bioscience), was added for 15 min at room temperature in the dark. Samples were then analysed by flow cytometry (Gallios, Beckman Coulter, Brea, CA, USA) using Megamix-Plus SSC beads (0.5, 0.9, and 3 µm, BioCytex, Marseille, France) to define the analysis gate. Representative images of flow cytometry workflow are reported in Supplemental Figure S1.

### 2.9. Statistical Analysis

Continuous variables were summarized as mean ± standard deviation (SD), while categorical variables were summarized as frequency (n) and percentage (%). Patient characteristic continuous variables were analysed by Student’s t-test, while discrete ones were analysed by Fisher’s exact test. Plasma biomarkers were first compared between controls and MVP patients by general linear models. As there were major differences in the clinical features in the study groups, three statistical models with different levels of adjustment for baseline clinical features were employed: Model 1, unadjusted; Model 2, adjusted for body mass index; and Model 3, adjusted for body mass index and treatment with anti-hypertensive drugs. To evaluate differences between pre- and post-operative MVP biological variables, a multivariate model taking into account repeated measurements was implemented. All the analyses were performed using IBM SPSS Statistic v25 (International Business Machines Corporation, New York, NY, USA), and images were created with Graphpad Prism v7.0 (GraphPad Software, San Diego, CA, USA).

## 3. Results

### 3.1. Patient Characteristics

Demographic, laboratory, and drug therapies of the study population are listed in Table 1. Body mass index was significantly lower in MVP patients than in controls, as reported by other authors [2]. In addition, a significant difference in the use of beta-blockers between the two groups was reported (*p* = 0.001).

As expected, the qualitative and quantitative pre-operative echocardiographic characteristics (Table 2), such as the left ventricular diastolic and the systolic volumes, were significantly increased in MVP patients compared to controls (both *p* < 0.001). Likewise, the left atrial area and the pulmonary artery systolic pressure were significantly greater in MVP patients than in controls (*p* < 0.001), while the ejection fraction (LVEF) was comparable between the two groups. In addition, most MVP patients presented chordal rupture that is a near universal finding in this class of patients [34]. The post-operative echocardiographic characteristics were significantly reduced when compared to the pre-operative ones (*p* < 0.01); however, all post-operative variables besides pulmonary artery systolic pressure remained significantly higher than those measured in controls (*p* < 0.05), with the exception of LVEF. Overall, 98% of patients (*n* = 44) underwent successful MVRep (i.e., residual regurgitation less than mild). This high percentage of successful repair prevented us from making any meaningful comparison with patients with residual mitral regurgitation.

In Supplemental Table S1 are depicted the surgical procedural characteristics and post-operative clinical data of the MVP patients included in this study.

### 3.2. Oxidative Stress Status Assessment and Antioxidant Defence System Evaluation

**Figure 1:**
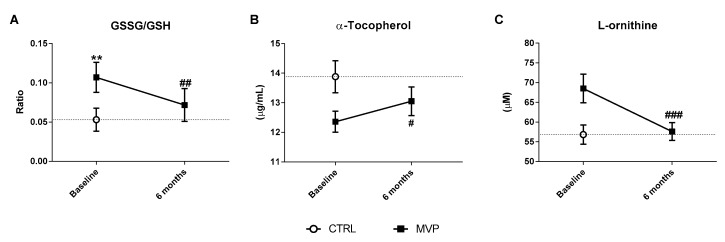
Oxidative stress status evaluation. (**A**) Oxidative stress index (GSSG/GSH) ratio in whole blood, (**B**) α-tocopherol, and (**C**) L-ornithine levels in plasma. All the analyses were performed in mitral valve prolapse (MVP) patients (*n* = 45) before (baseline) and 6 months after surgery and in controls (CTRL, *n* = 29) at the time of the visit at the clinic (CCM). Data are reported as mean ± 95% confidential interval. ** *p* ≤ 0.01 vs. CTRL; ### *p* ≤ 0.001, ## *p* ≤ 0.01, and # *p* ≤ 0.05 vs. MVP at baseline.

The evaluation of oxidative stress status has been performed by the assessment of GSSG/GSH ratio [27]. At baseline, the levels of this recognized oxidative stress index were significantly higher in MVP than in controls (*p* = 0.001—Table 3). Six months after the intervention, we found that this ratio was significantly decreased in MVP patients (0.11 ± 0.06 at baseline vs. 0.07 ± 0.07 at 6 months post surgery, *p* = 0.008—Figure 1A). In addition, αT, the main vitamin E form involved in the thiol redox cycle, which involves recycling GSH intracellular concentrations [35], showed a trend toward a reduction in MVP patients at baseline compared to controls (*p* = 0.056—Table 3).

Six months post-surgery, we noticed a significant increment of αT levels in MVP patients compared to their baseline (12.4 ± 2.4 vs. 13.1 ± 3.3 µg/mL, respectively; *p* = 0.05), becoming similar to αT levels in controls (13.9 ± 2.9 µg/mL—Figure 1B).

In parallel to glutathione and vitamin E evaluation, we also evaluated Orn levels, the product of arginase enzyme, whose activity is upregulated by a high oxidative status [36]. MVP patients, compared to controls, showed a trend toward an increment in Orn levels (*p* = 0.141—Table 3). Six months post surgery, Orn concentrations significantly dropped compared to baseline (68.5 ± 24.4 µM vs. 57.6 ± 15.2 µM, respectively; *p* = 0.001), being similar to those measured in controls (56.8 ± 13.0 µM—Figure 1C).

### 3.3. Endothelial Function Evaluation

Circulating levels of OPG, ADMA, SDMA, GABR, γT, and EMPs were used as surrogate markers of endothelial function.

**Figure 2:**
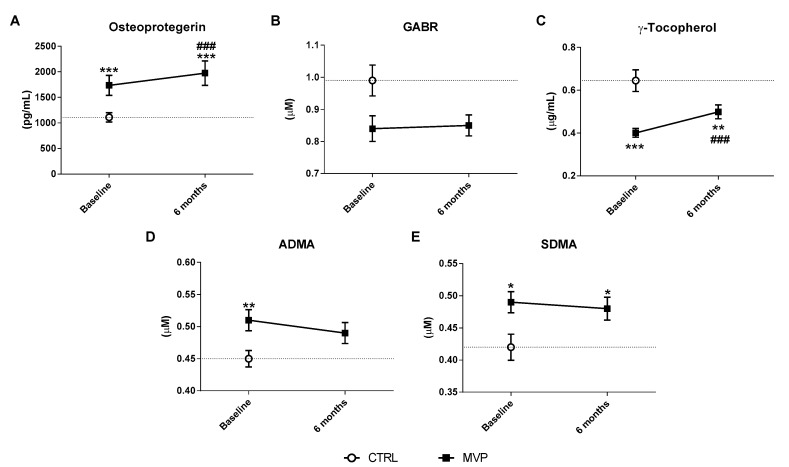
Endothelial function assessment. Plasma levels of (**A**) osteoprotegerin (OPG), (**B**) the index of global L-arginine bioavailability (GABR), (**C**) γ-tocopherol, (**D**) asymmetric (ADMA), and (**E**) symmetric (SDMA) dimethylarginines. All the analyses were performed in mitral valve prolapse (MVP) patients (*n* = 45) before and 6 months after surgery and in controls (CTRL, *n* = 29). Data are reported as mean ± 95% confidential interval. *** *p* ≤ 0.001, ** *p* ≤ 0.01, and * *p* ≤ 0.05 vs. CTRL; ### *p* ≤ 0.001 vs. MVP at baseline.

At baseline, OPG levels were significantly higher in MVP patients than in controls (*p* < 0.0001—Table 3 and Figure 2A), while GABR showed a trend toward a reduction in MVP when compared to controls (*p* = 0.126—Table 3, Table S2, and Figure 2B). γT was significantly lower in MVP than in controls (*p* < 0.0001—Table 3 and Figure 2C). In addition, both ADMA and SDMA were significantly increased in patients compared to controls (both *p* < 0.01—Table 3 and Figure 2D,E). At 6th month post surgery, OPG (post-op: 1974 ± 800.1 vs. pre-op: 1734 ± 653.3 pg/mL; *p* < 0.0001—Figure 2A) and γT levels (post-op: 0.50 ± 0.22 vs. pre-op: 0.40 ± 0.14 µg/mL; *p* = 0.001—Figure 2C) significantly increased when compared to their baseline. GABR index (post-op: 0.85 ± 0.27 vs. pre-op: 0.84 ± 0.27 µM—Figure 2B and Table S2), ADMA (post-op: 0.49 ± 0.11 vs. pre-op: 0.51 ± 0.11 µM—Figure 2D), and SDMA (post-op: 0.48 ± 0.12 vs. pre-op: 0.49 ± 0.11 µM—Figure 2E) concentrations, measured at the same time point, remained comparable to their baseline.

**Figure 3:**
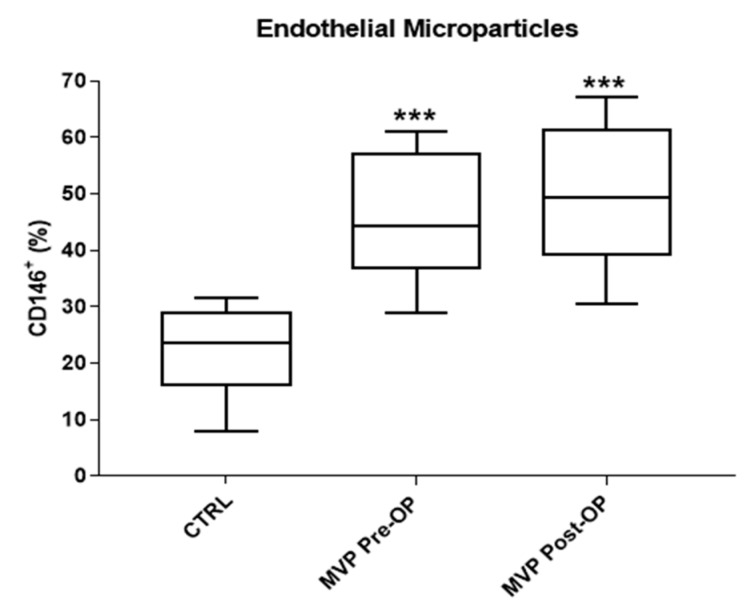
Plasma circulating CD146+ endothelial microparticles (EMPs). The central line illustrates the median, while box limits indicate the 25th and 75th percentiles. All the analyses were performed in a subgroup of mitral valve prolapse (MVP) patients before (Pre-operative, Pre-OP, *n* = 10) and 6 months (Post-operative, Post-OP, *n* = 10) after surgery and in a subgroup of controls (CTRL, *n* = 10). *** *p* ≤ 0.001 vs. CTRL.

Finally, we evaluated plasma EMPs at both time points. At baseline, the percentage of circulating EMPs was significantly higher in MVP patients than in controls (45.7 ± 11.1% vs. 22.5 ± 7.5%, respectively; *p* < 0.001—Figure 3) and did not change six months after surgery (49.7 ± 12.5% vs. 45.7 ± 11.1%, respectively—Figure 3). Overall, these results suggest the presence of an endothelial dysfunction that is not reverted by surgical intervention.

## 4. Discussion

To the best of our knowledge, this is the first study that evaluates systemic oxidative stress status and endothelial function before and 6 months after surgical intervention in patients with MVP. Our results showed that the increased oxidative stress levels detected before surgery significantly diminished 6 months after the intervention. In contrast, the impairment of vascular function persists even after valve repair, suggesting a putative link between endothelial dysfunction and MVP aetiology.

MVP with severe MR is the most common cause of mitral valve surgery and one of the most frequent valve diseases in adults [2]. In this pathological contest, data concerning systemic oxidative stress status and endothelial dysfunction are limited [13]. Chen et al. [37] identified a possible link between serum oxidative stress index; atrial contractile dysfunction; and, consequently, atrial fibrillation in human severe MR.

Experiments performed in canine myxomatous mitral valve disease suggested increased oxidative stress levels in both plasma and tissues, and endothelial function impairment [38,39]. In this dog model, genetically predisposed to MR development, the association between mitral valve disease and declining vascular endothelial function has been confirmed [40].

An important antioxidant system in humans is represented by αT and Γt, which are the two main forms of vitamin E [35,41]. αT is mainly involved in glutathione recycling, while γT has been shown to limit the endothelial dysfunction caused by hyperglycemia [42]. In our study, at baseline, both αT and γT were lower in MVP patients compared to controls. Six months after surgery, αT concentrations returned to levels comparable to controls, while γT levels remained lower. These results are in accordance with the ability of mitral valve surgery to completely restore the oxidative stress imbalance in MVP patients, without improving the endothelial function.

Endothelial dysfunction is now recognized as one of the main triggers for cardiovascular diseases. Several factors are involved in the development of this phenomenon and, among them, the bioavailability of Arg. Arginine is the common substrate for both nitric oxide synthase (NOS) and arginase enzymes, being the sole nitrogen source for NO synthesis [43]. Hence, the counteracting function of arginase enzyme in NO production and in NO-mediated vasodilatory function plays a key role. Indeed, arginase, an enzyme that consumes Arg to produce Orn, is increased in inflammatory and oxidative stress conditions [44]. The high Orn levels found in MVP patients support the concept of compromised endothelial function and the altered oxidative stress status in MVP patients.

The use of GABR could be an indirect comprehensive marker of the NO biosynthetic pathway, since it takes into account both the levels of the substrate (Arg) and of its major catabolic products (Orn and Cit) in vivo [45]. Indeed, diminished GABR is associated with both development of atherosclerosis and long-term risk for major adverse cardiac events [45]. Other key players in NO synthesis impairment are represented by two dimethylarginines, namely, SDMA and ADMA. SDMA can inhibit Arg uptake by blocking the cationic amino acid transporters [46], while ADMA is the major endogenous inhibitor of NOS [47]. The presence of increased levels of SDMA six months post surgery further supports the hypothesis of the inability of MVRep to completely resolve the endothelial dysfunction in MVP patients.

Our results showed an increased number of EMPs in MVP patients before surgery, and that these levels remained high even after MVRep. In line with this, an increased number of plasma EMPs found in MVP patients leads to Akt/endothelial nitric oxide synthase (eNOS)-HSP90 signaling pathway inhibition and therefore to mitral valve endothelial dysfunction [14].

Finally, we have previously shown that OPG is involved in the endothelial-to-mesenchymal transition of endothelial cells isolated from MVP patients [21]. Hence, it could be used as a surrogate marker of activated endothelial cells prior or during endothelial damage. Our results showed that OPG levels did not decrease after surgical intervention; rather, they continued to rise.

Some potential limitations of our study need to be discussed. The number of patients is exiguous, and these data need to be validated in large cohorts. This study is focused only on circulating markers, and no functional or tissue measurements were performed. In addition, control subjects were evaluated only at baseline and not followed up; nevertheless, in healthy subjects, the evaluated markers are quite stable, thus, it could be inferred that no changes occurred in this short period. The follow-up at 6 months could be a short window. However, systemic inflammation is resolved one-month post surgery; thus, the systemic alteration caused by the cardiac surgery should have not influenced the results [48]. Finally, circulating markers of oxidative stress and endothelial dysfunction could overlap. However, our analysis included more than one marker already linked to oxidative stress or endothelial dysfunction.

## 5. Conclusions

Overall, the results obtained in this study highlight the ability of mitral valve surgery to completely restore the increased oxidative stress levels evidenced in MVP patients. Conversely, the impairment in endothelial function still present six months after surgical procedure draws special attention to the need of studies aimed to dissect the molecular mechanisms of MVP.

In conclusion, our data suggest that endothelial dysfunction is not resolved by mitral valve surgery. Further studies aimed to highlight the presence of a causal link between endothelial function and mitral valve prolapse have to be conducted in order to define specific pharmacological treatments able to prevent disease progression.

## Figures and Tables

**Table 1:** Patient characteristics.

Variable	Control (*n* = 29)	MVP (*n* = 45)	*p*-Value
Age (years)	57.3 ± 11.2	61.0 ± 11.5	0.18
Sex (male)	19 (65.5%)	31 (69%)	0.80
Diabetes, n (%)	3 (10.3%)	4 (8.9%)	1.00
Hypertension, n (%)	14 (48.3%)	17 (37.8%)	0.47
Dyslipidemia, n (%)	14 (48.3%)	27 (60.0%)	0.35
Smokers, n (%)	6 (20.7%)	5 (11.1%)	0.32
BMI (Kg/m^2^)	27.2 ± 4.0	24.8 ± 2.8	**0.003**
Total Cholesterol (mg/dL)	215.4 ± 38.8	214.1 ± 46.8	0.90
Triglycerides (mg/dL)	113.4 ± 48.1	109.1 ± 43.1	0.69
HDL (mg/dL)	55.7 ± 16.6	51.1 ± 13.0	0.19
LDL (mg/dL)	137.0 ± 37.4	132.0 ± 44.9	0.62
**Drug Therapies**			
Antiplatelets, n (%)	1 (3.4%)	3 (6.7%)	1.00
Angiotensin receptor blockers, n (%)	4 (13.8%)	3 (6.7%)	0.42
Converting enzyme inhibitors, n (%)	5 (17.2%)	15 (33.3%)	0.18
Calcium channel blockers, n (%)	3 (10.3%)	3 (6.7%)	0.67
Beta-blockers, n (%)	1 (3.4%)	17 (37.8%)	**0.001**
Nitrates, n (%)	0 (0%)	1 (2.2%)	1.00
Statins, n (%)	6 (20.7%)	7 (15.6%)	0.76

BMI: body mass index; HDL: high-density lipoprotein; and LDL: low-density lipoprotein. Continuous variables are represented as mean ± SD.

**Table 2:** Echocardiographic data of control subjects and patients that underwent mitral valve repair.

	Control (*n* = 29)	MVP (*n* = 45)	
Variable		Pre-Operative	Post-Operative (6 Months)
LVEF, %	66.6 ± 5.7	62.9 ± 9.8	58.0 ± 7.6 ^#,§^
Left Ventricular Diastolic Volume, mL	79.5 ± 23	137.5 ± 30.9 *	100.7 ± 30.4 ^#,§^
Left Ventricular Systolic Volume, mL	26.2 ± 8.1	51.6 ± 20.2 *	43.8 ± 19.1 ^#,§^
Left Atrial Area, cm^2^	18.4 ± 4.0	29.8 ± 8.4 *	25.7 ± 5.6 ^#,§^
PAP, mmHg	27.2 ± 4.3	34.1 ± 8.3 *	28.8 ± 6.2 ^#^
EROA, cm^2^	-	0.6 ± 0.2	-
MAC (%)	-	8 (18)	-
Chordal Rupture (%)	-	32 (71)	-

LVEF: left ventricular ejection fraction; PAP: pulmonary artery systolic pressure; EROA: effective regurgitant orifice area; and MAC: mitral annulus calcification. Continuous variables are represented as mean ± SD. * *p* < 0.001 vs. controls; # *p* < 0.01 vs. pre-operative; and § *p* < 0.05 vs. controls.

**Table 3:** Circulating biomarkers.

Oxidative Stress	Model ^a^	Control (*n* = 29)	MVP (*n* = 45)	*p*-Value
GSSG/GSH	1	0.05 (0.02, 0.07)	0.11 (0.09, 0.13)	**<0.0001**
	2	0.04 (0.02, 0.07)	0.11 (0.09, 0.13)	**<0.0001**
	3	0.04 (0.01, 0.07)	0.12 (0.09, 0.14)	**0.001**
α-tocopherol	1	13.87 (12.91, 14.84)	12.35 (11.58, 13.12)	**0.017**
	2	13.8 (12.8, 14.8)	12.4 (11.6, 13.19)	**0.048**
	3	13.59 (12.55, 14.64)	12.53 (11.71, 13.35)	0.056
L-ornithine	1	56.84 (49.16, 64.51)	68.51 (62.35, 74.67)	**0.021**
	2	57.26 (49.29, 65.23)	68.24 (61.91, 74.57)	0.064
	3	57.42 (48.98, 65.87)	68.13 (61.54, 74.72)	0.141
**Endothelial function**	**Model ^a^**	**Control (*n* = 29)**	**MVP (*n* = 45)**	***p*-Value**
Osteoprotegerin	1	1109.3 (912.0, 1306.5)	1733.6 (1575.2, 1891.9)	**<0.0001**
	2	1111.34 (906.1, 1316.58)	1732.26 (1569.36, 1895.17)	**<0.0001**
	3	1143.97 (928.08, 1359.86)	1711.24 (1542.68, 1879.8)	**<0.0001**
GABR	1	0.99 (0.89, 1.09)	0.83 (0.75, 0.91)	**0.020**
	2	0.98 (0.88, 1.09)	0.83 (0.75, 0.92)	0.068
	3	1 (0.89, 1.1)	0.83 (0.74, 0.91)	0.126
γ-tocopherol	1	0.64 (0.57, 0.71)	0.4 (0.34, 0.46)	**<0.0001**
	2	0.64 (0.56, 0.71)	0.4 (0.34, 0.46)	**<0.0001**
	3	0.63 (0.55, 0.72)	0.4 (0.34, 0.46)	**<0.0001**
ADMA	1	0.44 (0.41, 0.48)	0.51 (0.48, 0.54)	**0.006**
	2	0.45 (0.41, 0.49)	0.5 (0.48, 0.53)	**0.009**
	3	0.47 (0.43, 0.5)	0.49 (0.47, 0.52)	**0.001**
SDMA	1	0.42 (0.38, 0.46)	0.49 (0.45, 0.52)	**0.016**
	2	0.41 (0.37, 0.45)	0.49 (0.46, 0.53)	**0.011**
	3	0.47 (0.43, 0.5)	0.49 (0.47, 0.52)	**0.009**

Continuous variables are represented as mean and 95% confidence interval. ^a^ Model 1 = unadjusted; Model 2 = adjusted for body mass index; and Model 3 = adjusted for body mass index and treatment with anti-hypertensive drugs.

## Supplementary Materials

The following are available online at https://www.mdpi.com/2077-0383/8/6/835/s1, Figure S1: Flow cytometry workflow, Table S1: Surgical procedural characteristics and post-operative clinical data of patients that underwent mitral valve repair, and Table S2: Circulating plasma levels of Arginine and Citrulline.

## References

[1] Delling F.N., Vasan R.S. (2014). Epidemiology and pathophysiology of mitral valve prolapse: New insights into disease progression, genetics, and molecular basis. Circulation.

[2] Freed L.A., Levy D., Levine R.A., Larson M.G., Evans J.C., Fuller D.L., Lehman B., Benjamin E.J. (1999). Prevalence and clinical outcome of mitral-valve prolapse. N. Engl. J. Med..

[3] Singh R.G., Cappucci R., Kramer-Fox R., Roman M.J., Kligfield P., Borer J.S., Hochreiter C., Isom O.W., Devereux R.B. (2000). Severe mitral regurgitation due to mitral valve prolapse: Risk factors for development, progression, and need for mitral valve surgery. Am. J. Cardiol..

[4] Sampaio R.O., Grinberg M., Leite J.J., Tarasoutchi F., Chalela W.A., Izaki M., Spina G.S., Rossi E.G., Mady C. (2005). Effect of enalapril on left ventricular diameters and exercise capacity in asymptomatic or mildly symptomatic patients with regurgitation secondary to mitral valve prolapse or rheumatic heart disease. Am. J. Cardiol..

[5] Supino P.G., Khan N., Hai O., Herrold E.M., Hochreiter C., Borer J.S. (2014). Relation of indirect vasodilator use to prognosis in patients with chronic severe mitral regurgitation. Cardiology.

[6] Varadarajan P., Joshi N., Appel D., Duvvuri L., Pai R.G. (2008). Effect of beta-blocker therapy on survival in patients with severe mitral regurgitation and normal left ventricular ejection fraction. Am. J. Cardiol..

[7] Ahmed M.I., Aban I., Lloyd S.G., Gupta H., Howard G., Inusah S., Peri K., Robinson J., Smith P., McGiffin D.C. (2012). A randomized controlled phase iib trial of beta(1)-receptor blockade for chronic degenerative mitral regurgitation. J. Am. Coll. Cardiol..

[8] Lang R.M., Adams D.H. (2012). 3d echocardiographic quantification in functional mitral regurgitation. JACC Cardiovasc. Imaging.

[9] Nishimura R.A., Otto C. (2014). 2014 acc/aha valve guidelines: Earlier intervention for chronic mitral regurgitation. Heart.

[10] Nishimura R.A., Otto C.M., Bonow R.O., Carabello B.A., Erwin J.P., Guyton R.A., O’Gara P.T., Ruiz C.E., Skubas N.J., Sorajja P. (2014). 2014 aha/acc guideline for the management of patients with valvular heart disease: Executive summary: A report of the american college of cardiology/american heart association task force on practice guidelines. Circulation.

[11] Baumgartner H., Falk V., Bax J.J., De Bonis M., Hamm C., Holm P.J., Iung B., Lancellotti P., Lansac E., Munoz D.R. (2017). 2017 esc/eacts guidelines for the management of valvular heart disease. Eur. Heart J..

[12] Shapero K., Wylie-Sears J., Levine R.A., Mayer J.E., Bischoff J. (2015). Reciprocal interactions between mitral valve endothelial and interstitial cells reduce endothelial-to-mesenchymal transition and myofibroblastic activation. J. Mol. Cell Cardiol..

[13] Cavalca V., Tremoli E., Porro B., Veglia F., Myasoedova V., Squellerio I., Manzone D., Zanobini M., Trezzi M., Di Minno M.N. (2013). Oxidative stress and nitric oxide pathway in adult patients who are candidates for cardiac surgery: Patterns and differences. Interact. Cardiovasc. Thorac. Surg..

[14] Ci H.B., Ou Z.J., Chang F.J., Liu D.H., He G.W., Xu Z., Yuan H.Y., Wang Z.P., Zhang X., Ou J.S. (2013). Endothelial microparticles increase in mitral valve disease and impair mitral valve endothelial function. Am. J. Physiol. Endocrinol. Metab..

[15] Dignat-George F., Boulanger C.M. (2011). The many faces of endothelial microparticles. Arterioscler. Thromb. Vasc. Biol..

[16] Guthrie R.B., Edwards J.E. (1976). Pathology of the myxomatous mitral value. Nature, secondary changes and complications. Minn. Med..

[17] Hulin A., Deroanne C., Lambert C., Defraigne J.O., Nusgens B., Radermecker M., Colige A. (2013). Emerging pathogenic mechanisms in human myxomatous mitral valve: Lessons from past and novel data. Cardiovasc. Pathol..

[18] Hulin A., Deroanne C.F., Lambert C.A., Dumont B., Castronovo V., Defraigne J.O., Nusgens B.V., Radermecker M.A., Colige A.C. (2012). Metallothionein-dependent up-regulation of tgf-beta2 participates in the remodelling of the myxomatous mitral valve. Cardiovasc. Res..

[19] Sainger R., Grau J.B., Branchetti E., Poggio P., Seefried W.F., Field B.C., Acker M.A., Gorman R.C., Gorman J.H., Hargrove C.W. (2012). Human myxomatous mitral valve prolapse: Role of bone morphogenetic protein 4 in valvular interstitial cell activation. J. Cell Physiol..

[20] Songia P., Porro B., Chiesa M., Myasoedova V., Alamanni F., Tremoli E., Poggio P. (2017). Identification of patients affected by mitral valve prolapse with severe regurgitation: A multivariable regression model. Oxid. Med. Cell. Longev..

[21] Songia P., Branchetti E., Parolari A., Myasoedova V., Ferrari G., Alamanni F., Tremoli E., Poggio P. (2016). Mitral valve endothelial cells secrete osteoprotegerin during endothelial mesenchymal transition. J. Mol. Cell Cardiol..

[22] Dal-Bianco J.P., Aikawa E., Bischoff J., Guerrero J.L., Handschumacher M.D., Sullivan S., Johnson B., Titus J.S., Iwamoto Y., Wylie-Sears J. (2009). Active adaptation of the tethered mitral valve: Insights into a compensatory mechanism for functional mitral regurgitation. Circulation.

[23] Wylie-Sears J., Aikawa E., Levine R.A., Yang J.H., Bischoff J. (2011). Mitral valve endothelial cells with osteogenic differentiation potential. Arterioscler. Thromb. Vasc. Biol..

[24] Vahanian A., Alfieri O., Andreotti F., Antunes M.J., Baron-Esquivias G., Baumgartner H., Borger M.A., Carrel T.P., De Bonis M., Evangelista A. (2012). Guidelines on the management of valvular heart disease (version 2012): The joint task force on the management of valvular heart disease of the european society of cardiology (esc) and the european association for cardio-thoracic surgery (eacts). Eur. J. Cardiothorac. Surg..

[25] Pepi M., Tamborini G., Maltagliati A., Galli C.A., Sisillo E., Salvi L., Naliato M., Porqueddu M., Parolari A., Zanobini M. (2006). Head-to-head comparison of two- and three-dimensional transthoracic and transesophageal echocardiography in the localization of mitral valve prolapse. J. Am. Coll Cardiol..

[26] Zoghbi W.A., Adams D., Bonow R.O., Enriquez-Sarano M., Foster E., Grayburn P.A., Hahn R.T., Han Y., Hung J., Lang R.M. (2017). Recommendations for noninvasive evaluation of native valvular regurgitation: A report from the american society of echocardiography developed in collaboration with the society for cardiovascular magnetic resonance. J. Am. Soc. Echocardiogr..

[27] Asensi M., Sastre J., Pallardo F.V., Lloret A., Lehner M., Garcia-de-la Asuncion J., Vina J. (1999). Ratio of reduced to oxidized glutathione as indicator of oxidative stress status and DNA damage. Methods Enzymol..

[28] Squellerio I., Caruso D., Porro B., Veglia F., Tremoli E., Cavalca V. (2012). Direct glutathione quantification in human blood by lc-ms/ms: Comparison with hplc with electrochemical detection. J. Pharm. Biomed. Anal..

[29] Werba J.P., Cavalca V., Veglia F., Massironi P., De Franceschi M., Zingaro L., Tremoli E. (2007). A new compound-specific pleiotropic effect of statins: Modification of plasma gamma-tocopherol levels. Atherosclerosis.

[30] Perez de Ciriza C., Lawrie A., Varo N. (2015). Osteoprotegerin in cardiometabolic disorders. Int. J. Endocrinol..

[31] Squellerio I., Tremoli E., Cavalca V. (2011). Quantification of arginine and its metabolites in human erythrocytes using liquid chromatography-tandem mass spectrometry. Anal. Biochem..

[32] Morris C.R., Kato G.J., Poljakovic M., Wang X., Blackwelder W.C., Sachdev V., Hazen S.L., Vichinsky E.P., Morris S.M., Gladwin M.T. (2005). Dysregulated arginine metabolism, hemolysis-associated pulmonary hypertension, and mortality in sickle cell disease. JAMA.

[33] Sourij H., Meinitzer A., Pilz S., Grammer T.B., Winkelmann B.R., Boehm B.O., Marz W. (2011). Arginine bioavailability ratios are associated with cardiovascular mortality in patients referred to coronary angiography. Atherosclerosis.

[34] Roberts W.C., Vowels T.J., Ko J.M., Hebeler R.F. (2014). Gross and histological features of excised portions of posterior mitral leaflet in patients having operative repair of mitral valve prolapse and comments on the concept of missing (= ruptured) chordae tendineae. J. Am. Coll. Cardiol..

[35] Packer L., Weber S.U., Rimbach G. (2001). Molecular aspects of alpha-tocotrienol antioxidant action and cell signalling. J. Nutr..

[36] Thengchaisri N., Hein T.W., Wang W., Xu X., Li Z., Fossum T.W., Kuo L. (2006). Upregulation of arginase by h2o2 impairs endothelium-dependent nitric oxide-mediated dilation of coronary arterioles. Arterioscler. Thromb. Vasc. Biol..

[37] Chen M.C., Chang J.P., Liu W.H., Yang C.H., Chen C.J., Fang C.Y., Hsieh Y.K., Wang Y.H., Chang H.W. (2009). Increased serum oxidative stress in patients with severe mitral regurgitation: A new finding and potential mechanism for atrial enlargement. Clin. Biochem..

[38] Olsen L.H., Mortensen K., Martinussen T., Larsson L.I., Baandrup U., Pedersen H.D. (2003). Increased nadph-diaphorase activity in canine myxomatous mitral valve leaflets. J. Comp. Pathol..

[39] Reimann M.J., Haggstrom J., Mortensen A., Lykkesfeldt J., Moller J.E., Falk T., Olsen L.H. (2014). Biopterin status in dogs with myxomatous mitral valve disease is associated with disease severity and cardiovascular risk factors. J. Vet. Intern. Med..

[40] Moesgaard S.G., Klostergaard C., Zois N.E., Teerlink T., Molin M., Falk T., Rasmussen C.E., Luis Fuentes V., Jones I.D., Olsen L.H. (2012). Flow-mediated vasodilation measurements in cavalier king charles spaniels with increasing severity of myxomatous mitral valve disease. J. Vet. Intern. Med..

[41] Christen S., Woodall A.A., Shigenaga M.K., Southwell-Keely P.T., Duncan M.W., Ames B.N. (1997). Gamma-tocopherol traps mutagenic electrophiles such as no(x) and complements alpha-tocopherol: Physiological implications. Proc. Natl. Acad. Sci. USA.

[42] Li Y., Bharath L.P., Qian Y., Ruan T., Anandh Babu P.V., Bruno R.S., Symons J.D., Jalili T. (2016). Gamma-carboxyethyl hydroxychroman, a metabolite of gamma-tocopherol, preserves nitric oxide bioavailability in endothelial cells challenged with high glucose. Exp. Biol. Med..

[43] Morris C.R., Poljakovic M., Lavrisha L., Machado L., Kuypers F.A., Morris S.M. (2004). Decreased arginine bioavailability and increased serum arginase activity in asthma. Am. J. Respir. Crit. Care Med..

[44] Yang Z., Ming X.F. (2013). Arginase: The emerging therapeutic target for vascular oxidative stress and inflammation. Front. Immunol..

[45] Tang W.H., Wang Z., Cho L., Brennan D.M., Hazen S.L. (2009). Diminished global arginine bioavailability and increased arginine catabolism as metabolic profile of increased cardiovascular risk. J. Am. Coll. Cardiol..

[46] Closs E.I., Basha F.Z., Habermeier A., Forstermann U. (1997). Interference of l-arginine analogues with l-arginine transport mediated by the y+ carrier hcat-2b. Nitric Oxide.

[47] Vallance P., Leone A., Calver A., Collier J., Moncada S. (1992). Accumulation of an endogenous inhibitor of nitric oxide synthesis in chronic renal failure. Lancet.

[48] Parolari A., Poggio P., Myasoedova V., Songia P., Pilozzi A., Alamanni F., Tremoli E. (2016). Molecular pathways activation in coronary artery bypass surgery: Which role for pump avoidance?. J. Cardiovasc. Med..

