# Supplementary material for: Endothelial Dysfunction in Patients with Severe Mitral Regurgitation
Source: J Clin Med. 2019 Jun 12;8(6):835. doi: 10.3390/jcm8060835 (PMC6616454; doi:10.3390/jcm8060835)
Supplement: Supplementary file 1 [file jcm-08-00835-s001.pdf]

Supplemental Figure S1. Flow cytometry workflow.

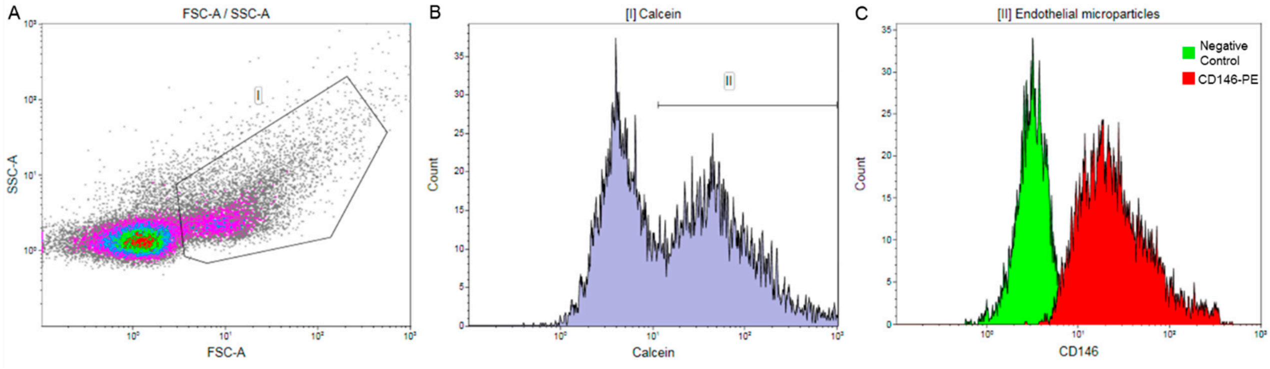

(**A**) Representative image of the scatter plot (Y axis: side scatter; X axis: forward scatter), reporting all acquired events. (**B**) Representative histogram showing events gated from [I], identified using Megamix-SSC Plus beads, and stained with calcein AM to detect intact microparticles. (**C**) Representative overlay of intact microparticles [II] unstained (negative control - green) and stained with a specific marker for endothelial released microparticles (CD146 - red) conjugated with phycoerythrin (PE).

Supplemental Table S1. Surgical procedural characteristics and post-operative clinical data of patients that underwent mitral valve repair.

**Variable**	**MVP** (n = 45)
Cardiopulmonary bypass time, min	114.7 ± 39.7
Aorta clamping time, min	92.2 ± 37.7
24h post-surgery – creatine kinase-MB, ng/mL	48.6 ± 25
Intensive-care stay, days	1.3 ± 0.7
24h post-surgery – atrial fibrillation, n (%)	16 (35.6)
At discharge – atrial fibrillation, n (%)	0 (0)
Pacemaker implantation, n (%)	2 (4.4)

Continuous variables are represented as mean ± SD.

Supplemental Table S2. Circulating plasma levels of Arginine and Citrulline.

	**Control** (n = 29)	**MVP** (n = 45)	
**Variable**		**Pre-operative**	**Post-operative (6 months)**
Arginine (μM)	80.8 ± 16.1	76.6 ± 18.2	70.8 ± 14.4 *
Citrulline (μM)	27.8 ± 7.9	29.8 ± 9.4	28.4 ± 6.6

Data are reported as mean ± SD. * $p < 0.05$ *vs* Controls-
